# Mathematical models and analysis tools for risk assessment of unnatural epidemics: a scoping review

**DOI:** 10.3389/fpubh.2024.1381328

**Published:** 2024-05-02

**Authors:** Ji Li, Yue Li, Zihan Mei, Zhengkun Liu, Gaofeng Zou, Chunxia Cao

**Affiliations:** ^1^Institute of Disaster and Emergency Medicine, Tianjin University, Tianjin, China; ^2^College of Management and Economics, Tianjin University, Tianjin, China

**Keywords:** unnatural epidemics, mathematical models, risk assessment, machine learning, analysis tools

## Abstract

Predicting, issuing early warnings, and assessing risks associated with unnatural epidemics (UEs) present significant challenges. These tasks also represent key areas of focus within the field of prevention and control research for UEs. A scoping review was conducted using databases such as PubMed, Web of Science, Scopus, and Embase, from inception to 31 December 2023. Sixty-six studies met the inclusion criteria. Two types of models (data-driven and mechanistic-based models) and a class of analysis tools for risk assessment of UEs were identified. The validation part of models involved calibration, improvement, and comparison. Three surveillance systems (event-based, indicator-based, and hybrid) were reported for monitoring UEs. In the current study, mathematical models and analysis tools suggest a distinction between natural epidemics and UEs in selecting model parameters and warning thresholds. Future research should consider combining a mechanistic-based model with a data-driven model and learning to pursue time-varying, high-precision risk assessment capabilities.

## 1 Introduction

Unnatural epidemics (UEs) are caused by human intervention and may be deliberate or accidental releases of naturally occurring or altered pathogens (1). UEs cause substantial harm due to their wide range of infectiousness, rapid transmission, insidious processes, and multiple transmission methods (2). The intricacy of crowd behaviors and uncertainty regarding time and geography in UE outbreaks make prevention and control difficult (3).

This emphasizes the importance of early identification, prediction, and warning in UEs (4). Mathematical models and analysis tools can help combat UEs in prediction, early warning, and risk assessment (5). A study has proposed a model for labeling abnormal outbreak patterns to predict the development trends of complex pathogens (6). To achieve automatic early warning and response to UEs, some scholars have proposed real-time surveillance and aberration detection algorithms for enhanced outbreak monitoring (7). In addition, analysis tools can use simple scoring rules to complete a risk assessment of UEs. Scholars can use the analysis tools to assess the needs of applied epidemiology and training programs to develop greater capacity (8) and rank highly hazardous microorganisms. To better integrate various mathematical models and analysis tools for disease prediction, early warning, and risk assessment, disease surveillance systems have emerged as practical application tools. Current disease surveillance systems require early warning mechanisms that can detect a significant increase in confirmed cases by analyzing historical data. However, these methods cannot estimate outbreak size or detect new UEs. The data on UEs are scarce, making projections less accurate. Existing mathematical models and analysis tools for prediction, early warning, and risk assessment of UEs lack comprehensive review.

Therefore, this study aims to address the following research questions: What kinds of mathematical models or analysis tools have been developed for risk assessment of UEs? How should the suitability of the mathematical models and analysis tools be verified? What is the application of mathematical models and analysis tools embedded in surveillance systems? By reviewing relevant literature, this study will provide insights into mathematical models and analysis tools used for quantifying the risk of UEs. The findings will assist public health officials in making timely projections and decisions about UEs.

## 2 Methods

### 2.1 Design

This scoping review was conducted using the recommended PRISMA-ScR (Preferred Reporting Items for Systematic Reviews and Meta-Analyses extension for Scoping Reviews) guidelines published in 2018 (9). Supplementary material S1 provides the PRISMA-ScR Checklist.

### 2.2 Information sources and search strategy

A rigorous literature search was conducted from inception to 31 December 2023 using various online databases, such as PubMed, Web of Science, Scopus, and Embase. A combination of three types of keywords for the issue is necessary: subjects (words such as bioterrorism), purposes (words such as predict), and methods (words such as model) (10). These search terms are limited to the title, abstract, and keywords of the articles. Logical operators “AND” and “OR” were used to combine terms to meet PubMed, Web of Science, Scopus, and Embase standards. Appropriate filters and keywords were selected for the four databases. Supplementary material S2 provides detailed search strategies.

### 2.3 Selection of studies

**Figure 1:**
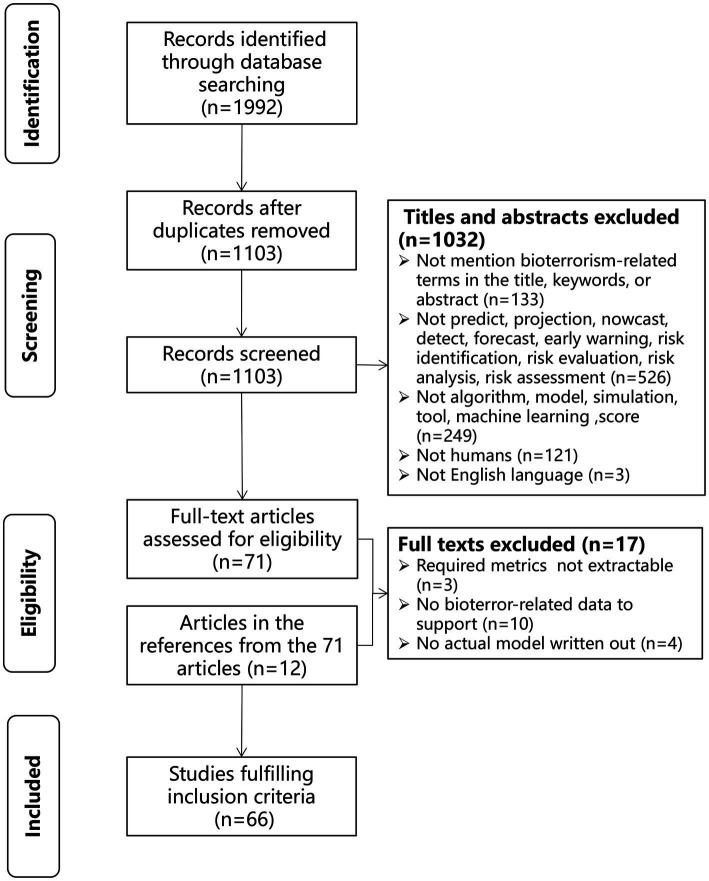
Flowchart depicting the study design.

All peer-reviewed sources were reviewed independently by two authors. A third author resolved disagreements. A total of 1992 articles were reviewed, and after de-duplication using Endnote X9 (Clarivate Analytics, United States), 1,103 articles met the nadir criteria to enter the data extraction stage. Seventy-one articles were screened for initial inclusion based on the constraints of the selected metrics in this study. Twelve articles were chosen using snowball sampling. Seventeen of the eighty-three articles were eliminated because the necessary metrics could not be derived. Finally, 66 articles were reviewed in this study (Figure 1). The study selection form is shown in Supplementary material S3.

### 2.4 Data extraction

Data were extracted from all identified studies using a predefined format. Variables included year of publication, country, first author, journal, and so on (10). Supplementary material S4 provides the data extraction form.

### 2.5 Characteristics of the included studies

Based on the current literature, risk assessment of UEs began to attract scholars from all fields in the 21st century, with more than 60% of the articles coming from the United States, which dominates research on mathematical models and analysis tools for risk assessment. Sixteen other countries have also published corresponding articles. Thirty-five journals contributed sixty-six articles to the study. With six articles, *Emerging Infectious Diseases*, *Morbidity and Mortality Weekly Report Supplements*, *and Risk Analysis* contributed the most articles. Supplementary material S5 summarizes the characteristics of the included studies. Supplementary material S6 displays the articles published in risk assessment studies using mathematical models and analysis tools.

### 2.6 Evidence synthesis

This scoping review used descriptive-analytical methods. The graphs were designed using R statistical software (version 4.1.3).

## 3 Categorizing of mathematical models and analysis tools for risk assessment of UEs

### 3.1 Data-driven models

Data-driven models are commonly used in statistical or machine learning methods such as support vector machines. Based on goodness-of-fit rather than mode-of-action or mechanism, data-driven models use correlations to find the optimal input variables to predict desired outputs. Therefore, their structure is “driven by the data” (11). Table 1 summarizes data-driven models for risk assessment of UEs.

**Table 1:** Summary of data-driven models for risk assessment of unnatural epidemics.

Model classes	Name of model	Data type*	Data (time or events)	Functions	Factors	Outcomes
Data-driven models	Function of mutual information (12)	AD	Influenza (2003–2006)	Early warning	Anti-influenza sales daily	Detection limit
	First-order model (13)	AD	ED* patient system data (1994–2003)	Risk identification	Age; gender; arrival/ discharge time and date; discharge diagnoses and disposition	Infectious disease effects; daily visit patterns
	Structural models (14)	HD	Counts of anthrax (2009–2010)	Risk prediction	Daily visits to care providers; counts of anthrax	Outbreak size
	Modified cyclical regression model (15)	AD	Ambulance calls (1993–2003)	Early detection	Ambulance dispatches; ILI* call types; number of virus isolates; temperature	Alarm threshold
	SVR* (16)	AD	Dengue outbreaks (2011–2015)	Early detection	Dengue cases; weather parameters	Short-term trend of dengue fever
	CUSUM* (17–19)	AD	Clinical datasets (2001–2002, 2005, 2012–2014)	Early warning	Chief complaints: count of each syndrome in each region	Daily counts
	FARR* (18)	AD	Clinical and non-clinical data (2005)	Early warning	Call types; discharge diagnosis; chief complaints; microbiology tests	Syndrome counts
	MA* (20, 21)	AD	Medical institutions and schools (2002–2003)	Early warning	Disease clusters; selected syndromes; personal information; ICD-9*; chief complaint; school absence rates; calls	Outbreak limits; event counts
	Bayesian approach (22)	SD	Anthrax attack (spread of anthrax)	Estimate the size and time of attack	Case counts; report timing	Attack size and time
	Bayesian aerosol release detector (23)	HD	ED* and meteorological data (1999–2005)	Estimate the size and time of attack	Complaint ED* visits vector; geography matrix; weather matrix	Location, quantity, time, and release probability
	Population-wide anomaly detection and assessment (24)	HD	Anthrax attack (spread of anthrax)	Estimate the size and time of attack	Chest X-ray or blood culture likelihood; Last ED* case; spore distribution; weather; location, height, date, and spore amount	Maximum joint detection time; patient count
	Bayesian networks (25)	AD	Simulated daily time series without outbreaks (1994–1999)	Early warning	Days of epidemic signal; day outbreak signal peaks; outbreak signal standard deviations above baseline; outbreak spike	Outbreak signals
	Bayesian approach (26)	HD	Sverdlovsk anthrax attack (1979)	Estimate the size, time, and dose of attack	Symptoms; incubation; attack rate; dose	Attack size, average dose; and time
	WSARE* algorithm (27, 28)	AD/ SD	Influenza B outbreak (2004) or epidemic data	Early detection	Clinic visit date; city code; ICD*; Age	Anomalous patterns
	Recursive least square adaptive filter (29–31)	AD	Urgent care, emergency, and polyclinic data (from 2002)	Estimate the size of attack	Chief complaints; demographic data	Number infected
	Space–time scan statistic (32–35)	HD	Outpatient data (spread of anthrax or respiratory)	Early warning	Patient visits/calls; count of each syndrome and zip code	Number of events
	Small area regression and testing (34)	AD	Outpatient data (spread of anthrax)	Early warning	ICD-9*; count of each syndrome	Number infected
	GLMM* (36)	AD	Electronic medical, demographic, and eligibility records (1996–2000)	Estimate infection probability	Individual measures; area or population measures; day descriptions; pre-surveillance days	Probability of a surveillance day case in an area
	Modified EWMA* (37)	AD	Clinical and non-clinical data (abnormal symptom attacks)	Early warning	Emergency room respiratory syndrome counts; office visit respiratory counts; OTC* influenza drug sales; school absence totals	Alarm threshold
	Seasonal ARIMA* (38)	HD	Syndromic surveillance systems data (2001–2003)	Estimate the size and time of attack	Respiratory syndromes; infected people; median incubation and prodromal times; proportion seeking prodromal care	Number infected
	Trimmed-mean seasonal models (39, 40)	HD	ED* data (1992–2002)	Estimate the size of attack	Daily visit totals	Number of visits
	Aggregate and local model (41)	HD	Hospital data (1998–2002)	Estimate the size of attack	Overall series mean; weekly signal; Yearly trend; chief complaint; ICD*	Daily visit totals
	Decision analytic model (42)	HD	Surveillance system data (2003) and simulated attack	Estimate the size and time of attack	Health state transition probability; utility values; cost estimates	Number of infected, lives, QALYs* and costs
	G- / P-Surveillance Methods (43)	AD	CDSC* (1997–2002)	Early detection	Brucellosis counts per week	Number of events
	CC and CI* (44, 45)	AD	Cryptosporidiosis outbreak (1997); Symptoms data (from 2001)	Early warning	NHSD* call data; incubation time distribution	Upper prediction limits for calls
	Recombinant temporal aberration detection algorithms (46)	AD	RESP*, GI* or other diseases (spread of RESP*, GI* or other diseases)	Early detection	Military clinic diagnosis; prescriptions; civilian doctor visits	Outbreak signals
	Supervised machine learning algorithms (47)	AD	Tweets in English (2017–2018)	Early detection	Tweet content; time of news story; first news article-related tweet or retweet; event-to-detection time	Number of tweets
	Risk analysis approach (48, 49)	SD	*Coccidioides immitis* and the anthrax attack (spread of *Bacillus anthracis* and Coccidioides immitis)	Estimate infection risk	Infectious inhalation dosage; expected exposure times; Infection threshold	Infection risk

*The following abbreviations are used. SD, Simulated data; AD, Authentic data; HD, Hybrid data (simulated data and authentic data); ILI, Influenza-like illness; EWMA, Exponentially weighted moving average; NA, No answer, used on forms; OTC, Over-the-counter; ICD-9, International Classification of Diseases, Ninth Revision; WSARE, What’s strange about recent events; ED, Emergency department; CDSC, Communicable disease surveillance center; GLMM, Generalized linear mixed models; MA, Moving average; CUSUM, Cumulative sum; ARIMA, Autoregressive integrated moving average model; CC and CI, Control chart and Confidence interval method; NHSD, National health service direct; RESP, Respiratory; GI, Gastrointestinal; FARR, Exceedance method by Farrington; QALYs, Quality-adjusted life years; SVR, Support vector regression.

#### 3.1.1 Application I: detect outbreaks

Data-driven models have important applications in prediction. The first application is to detect outbreaks of UEs. The function of mutual information (12) uses daily anti-influenza drug sales to determine detection limits (thresholds) of UEs to complete early warning. A first-order model (13) can identify outbreaks 1–2 weeks before respiratory disease events. This model uses patient monitoring data, including age, complaints, and discharge diagnoses. Structural model (14) predicts the outbreak level 2 weeks after the alert using real and simulated anthrax exposure counting with noise. This model can detect and predict partially observed epidemics. Modified cyclical regression model (15) uses ambulance dispatch data for outbreak detection to measure influenza mortality and report the alarm threshold. Support vector regression (16) links dengue cases with weather parameters and predicts 20-week dengue trends. Cumulative sum (CUSUM) method (17–19) and exceedance method by Farrington (FARR) (18) are often used for early warning. A “signal” is generated in the system when the observed and expected count disparities exceed a threshold. CUSUM is widely used to report the epidemic severity of regional outbreaks in a system. UEs can also be tracked by the moving average of numerous data cycles (20, 21). Furthermore, data-driven models can track public opinions during outbreaks. Using supervised machine learning algorithms (47), researchers can identify anthrax-related tweets. Plotting data over time helped determine if an event was detected (based on a spike in the number of tweets occurring three times). This shows that machine learning can use not only conventional clinical data but also public opinion data for outbreak detection.

#### 3.1.2 Application II: predict the size and time of attacks

The second application is directed at predicting the size and time of attacks. Bayesian methods and networks (22–26) can predict simulated anthrax attacks using case counts and attack time. Bayesian methods can quickly assess the extent and time of a biological error attack and predict how many people will develop symptoms and need medical care. The What’s Strange About Recent Events (WSARE) algorithm (27, 28) reports anomalous patterns based on time, location, and population at risk to construct an outbreak risk profile. The recursive least square adaptive filter (29–31) detects short-term outbreak signals because it emphasizes recent past data while calculating forecast counts. Space–time scan statistics (32–35) prioritize temporal and geographical partitions based on attack size and time. Furthermore, generalized linear mixed models (36) estimate the infection probability of being a case for a surveillance day in a setting area. Time series analysis, such as the modified exponentially weighted moving average technique (37), the seasonal autoregressive integrated moving average model (ARIMA) (38), trimmed-mean seasonal models (39, 40) and ARIMA (39), is frequently used to examine epidemic trends. It uses count data to predict outbreaks and detect abnormal epidemics early.

#### 3.1.3 Model performance

With the emergence of more and more mathematical models for the risk assessment of UEs, there is a growing focus on evaluating the performance of these models. Some studies (34, 41) use a single input data or multivariate aggregated data to check the sensitivity of the model prediction performance. The results prove that two-stream surveillance is superior to one-stream surveillance. Model performance can also be measured by model practice effectiveness. Decision-analytic model (42) predicts lives, quality-adjusted life years (QALYs), and costs for a series of simulated bioterrorist attacks. However, false positives of the model were also noticed. G-/P-surveillance methods (43) that use only incidence case count data set the time to 21–30 days or 1 year to detect a relatively sudden increase in incidence. It greatly reduces false alarms but decreases sensitivity. Control chart and confidence interval methods (44) use call data and incubation periods for early warning of outbreaks. In comparison, the control chart for false alarm control is stricter. On top of having case data, different models incorporate additional data based on factors affecting the issue. Recombinant temporal aberration detection algorithms (46) also employ outpatient diagnoses, volume, and other relevant factors to decompose existing temporal aberration detection algorithms into two consecutive phases. This method investigates the impact of each phase on outbreak detection performance by reporting outbreak signals. In addition, the algorithms enhance the model’s detection of UEs.

### 3.2 Mechanistic-based models

Mechanistic-based models use mechanisms and algorithms regardless of data availability (50, 51). Data are used to fit these mechanistic-based models and enable their operations. They aim to describe causation, although they typically contain empirical components as well (11). Table 2 summarizes mechanistic-based models for risk assessment of UEs.

**Table 2:** Summary of mechanistic-based models for risk assessment of unnatural epidemics.

Model classes	Name of model	Data type*	Data (time or events)	Functions	Factors	Outcomes
Mechanistic-based models	SIR* (52, 53)	SD	Synthetic population data (spread of smallpox)	Estimate the size of attack	Contact patterns; initial population size; initial infected population; susceptibility; relative infectivity; relative disease progression; relative mortality	Outbreak size
	SID* (54)	NA	Smallpox attack (spread of smallpox)	Estimate the size and time of attack	R_0_; incubation, prodromal, and symptomatic periods; initial-exposure cases; initial susceptibility; public health staff; vaccination count; processed daily per public health worker; contact count; maximum daily quarantine during symptoms	Total patients; duration; epidemic peak
	Back-calculation method (55)	HD	National statistics data (1991) and anthrax outbreaks	Estimate the size and time of attack	Initial cases; weather; population; travel patterns	Attack time, size, and location
	Markov chain model (56)	AD	Smallpox cases (1950–1971)	Estimate the size of attack	Numbers originally infected; transmission rate; daily quarantine and removal rate; intervention day; vaccine doses per case; Incubation time	Daily new-onset and cumulative cases; store vaccines
	Within-host mathematical model with spatial back-calculation method (57)	SD	Anthrax attack (spread of anthrax)	Risk identification and assessment	Previous antibiotic adherence; Postexposure vaccination; initial case delay range	Outbreak size
	Transmission model (58)	SD	Anthrax attacks (2006)	Estimate the size and time of attack	Personal data; behavior; social interactions	Patients and fatalities; vaccine dosages; eradication time
	Epidemic curve model (59)	AD	Sverdlovsk anthrax attack (1979)	Estimate the size of attack	Case numbers; incubation period	Outbreak size
	Dose- and time-dependent mathematical model (60)	AD	Bacillus tularemiatularensis dataset (1998)	Predict health effects	Fever incubation; fever onset; near-maximum body temperature	Disease incidence
	Simulation modeling (61)	SD	Anthrax attack (spread of anthrax)	Estimate the time interval of attack	Released spores; floor; air exchange rate; spore sedimentation; suspended spores; transfer processes	Spore dispersion time; aerosol removal time interval
	Wells-Riley mathematical model (62)	SD	Anthrax attack (spread of anthrax)	Risk identification	Environment; host; organism virulence	Infection risk
	Pathogen fate and transport model (63)	AD	Anthrax attacks (2001)	Estimate attack risk	Spore release; risk of sickness; spore setting velocity; resuspension rate; sample recovery efficiency	Mortality risk

*The following abbreviations are used. SD, Simulated data; AD, Authentic data; HD, Hybrid data (simulated data and authentic data); SID, Susceptible-infected-dead model; NA, No answer, used on forms; SIR, Susceptible-infected-recovered model.

Mechanistic-based models for risk assessment of UEs mainly rely on infectious disease transmission mechanisms to construct a skeleton-like framework and commonly include Susceptible-Exposed-Infected-Removed (SEIR) models (52, 54), disease propagation differential equations, and other models (64, 65). Unlike data-driven models, mechanistic-based models emphasize fitting the model to the data. Mechanistic-based models can predict the size of UEs. A probabilistic anthrax model (55) uses a Markov chain Monte Carlo sampling algorithm to estimate the outbreak size. It evaluates post-anthrax release mitigating measures to better estimate unnatural outbreaks. Most mechanistic-based models (53, 56–59) predict cumulative infections, daily numbers of infections, and the size of UEs based on pathogen transmission in the target population. These models create rapid reaction systems and procedures by identifying and geospatially analyzing UEs. A few mechanistic-based models focus on the impact of dose–response mechanisms on the risk assessment of UEs. Input indicators are crucial to dose–response models (48, 49, 60–63). The input indicators can be included in clinical indicators (e.g., human respiration rate), environmental indicators (e.g., indoor room area), and pathogen dispersal indicators (e.g., spore dispersal rate). Dose–response models can estimate the size and timing of UEs for airborne pathogen infections, using human, environmental, and pathogen data to assess infection risk. Dose–response models can provide a reliable reference for risk assessors and healthcare decision-makers.

### 3.3 Analysis tools

In addition to data-driven and mechanistic-based models, analysis tools are often applied to UEs. Analysis tools, including qualitative and quantitative tools, enable decision-makers to quickly and reliably differentiate between natural epidemics and UEs (66). Scores are assigned based on answers, similar to questionnaire scoring. Finally, these characteristics are scored to reflect the risk of UEs. Table 3 provides analysis tools for risk assessment of UEs.

**Table 3:** Summary of analysis tools for risk assessment of unnatural epidemics.

Tools	Functions	Factors	Outcomes
Epidemiologic clues (67)	Risk identification	Epidemiologic clues of unusual illness (rare agent/rare disease) or unusual patterns of person, place, and time; routine illnesses that some agents cause or infection with some agents	Possibility of bioterrorism
Original GFT* (68, 69)	Risk identification	Political, military, and social analysis of the crisis zone; pathogen traits; epidemic features; disease incidence; early-stage fever profile	Bioterrorism likelihood
New gradual model of bioterrorism risk assessment (70)	Define suspended perpetrators	Perpetrators; agents; means or media of delivery; targets	Score of suspended perpetrators
Scoring system for unusual epidemic events (71)	Risk identification	Cases; time and spatial distribution of cases	Possibility of a deliberate or accidental outbreak
Risk assessment matrix (72)	Risk assessment	Public health impact; suspected purposeful act; information source	Score of biological threats risk
Scoring method with 33 parameters (73)	Risk assessment	Infection/reservoir or pathogen; transmission, distribution, and biological agent target group	Bioterrorism likelihood
Modified GFT* (74–76)	Risk identification	Demographic data; location data; data of multiple risk factors	Bioterrorism likelihood
Radosavljevic-belojevic method (77)	Risk identification	Unusual biological cases; unusual epidemic spread; higher morbidity/mortality; abnormal contact; pathogen reservoir/perpetrator; pathogen/biological agent; delivery methods; target/vulnerable population	Outbreak score
Existing reclassification of potential biological weapons method (78)	Risk identification	Ease of use; virulence; mortality; person-to-person transmission; inoculation period; discreet clinical picture; laboratory diagnosis; treatment choices; environmental/animal impacts; disease chronicity; hospital burden; pathogen public perception	Score of biological threats risk
Generic risk ranking system (8)	Risk identification	History; agent accessibility; production and storability; diagnostics; agent dispersion; human and animal population countermeasures; public health impact; ecological and economic effects; panic risk	Risk ranking of agents
Bioweapon risk assessment tool (79, 80)	Risk assessment	Infectivity; infection-to-disease ratio; predictability (and incubation period); morbidity and mortality; ease of large-scale production and storage; aerosol stability; environmental stability; ease of dispersal; communicability; prophylactic countermeasure availability; therapeutic countermeasure availability; ease of detection	Score of biological threats risk
Assessment method for potential biological threat agents (81)	Risk identification	Public health impact; dissemination potential; Public perception; special preparation	Categories for potential biological threat agents

*The following abbreviations are used. GFT, Grunow–Finke assessment tool.

A qualitative tool (67) presents epidemiological clues that highlight features of an epidemic that may suggest an unnatural attack. Another class of quantitative tools (68–77, 79–81) can calculate the risk factor scores to differentiate between natural epidemics and UEs. If the risk factor scores exceed the threshold, the event is likely unnatural. Such a simple and useful scoring method enables rapid differentiation between biological attacks and other epidemics, shortens the time for decision-makers to report the epidemic situation, and develops appropriate responses. Some scoring tools (8, 78) also assess biological weapon danger.

## 4 Validation of mathematical models and analysis tools for risk assessment of UEs

Model validation checks if a “model reliably reproduces the crucial behavior and quantities of interest within the intended context of use” (82). Model validation enhances model performance and applicability. The general analytical perspective of model validation starts with model uncertainty. Model uncertainty requires further refinement or tests of the model itself to affect final results. The validity of models and tools is increased by calibration (12, 14), improvement (74–76), and comparison (13, 55, 59, 63) under model uncertainty. Table 4 shows the model validation.

**Table 4:** Validation of mathematical models and analysis tools for risk assessment of unnatural epidemics.

Type of model validation	Validation focus	Descriptions
Model calibration	Change the data volume (4)	Adapt the data monitoring area’s size or the variety of data sources
	Lessen noisy data (14)	Simulated outbreak is correctly isolated from “normal” background signal
	Influence of different model input/output on model results (17, 23, 26, 38, 42, 52, 54, 55, 59, 63)	Assess the sensitivity of modeling risk to different input uncertainties
Model improvement	Recalibration of tool (74–76)	Recalibration of the Grunow–Finke assessment tool
Model comparison	Comparison of different models (13, 25, 34, 42, 46, 47)	Comparison of algorithms and identification of suitable algorithms for use

### 4.1 Model calibration

Model calibration is a performance test of the model that has been built. Model calibration, which adjusts model parameters to match experimental data, affects a model’s ability to predict the future (83). This approach is particularly useful when a model involves multiple inputs or parameters (84). This study summarizes three directions for model validation.

First, it involves changing various inputs or model parameters (17, 23, 26, 38, 42, 52, 54, 55, 59, 63). Different model input–output combinations are tested to see how they affect results. Modeling risk sensitivity to input uncertainties is commonly studied. Then, method validation analyses the same scenario for different combinations of inputs and is assessed with evaluation indicators, such as R-squared, root mean square error (RMSE), mean square error (MSE), mean absolute error (MAE), mean absolute percentage error (MAPE), sensitivity, and receiver operating characteristic (ROC) curve. In addition to modifying model inputs and outputs, model misspecifications can affect model estimate accuracy. Legrand et al. (55) replicated the process of estimating anthrax spread in a model but intentionally misspecified parameter values, data, or model structure. The results suggest this can affect model estimation accuracy. Second, it involves reducing the influence of noisy data on genuine data. Background subtraction can test the multiple forward predictions of the established structural model (14). The final model was able to correctly isolate the simulated epidemic from the “normal” background signal. Third, it involves changing data sources to test model applicability. One approach (12) is to expand the monitored area by collecting data on daily sales of disease-related drugs. Researchers can then check drug purchase records for abnormalities to assess whether an epidemic has occurred. To test the sensitivity of the model’s prediction ability, another approach (34, 41) involves using a single input data set and multivariate aggregated data. Model calibration is common when a system is poorly understood or measured (83). However, model calibration is difficult because quantitative epidemic prediction models have many parameters to calibrate. Thus, poor parameter determination is the main obstacle to model prediction accuracy.

### 4.2 Model improvement

Model improvement extends beyond method validation. It involves calibrating the model to increase event risk assessment accuracy. This process typically leads to improved accuracy of a single model. The original technique is insensitive to unnatural infectious diseases and is weak at spotting previous epidemics with known causes. The Grunow-Finke tool (GFT) is the best-known tool for such differentiation. The recalibration of GFT for identifying UEs (74–76) involved removing criteria from the old GFT. In addition, it also adjusts weighting factors for additional criteria and reintroduces significant ones. It sets evaluation tools that identify the thresholds of natural epidemics and UEs, too. Model improvement focuses on the lower sensitivity and specificity of the model or tool to improve the broad applicability of them. Model improvement involves reviewing the original model’s assumptions and determining if existing changes conflict with existing data.

### 4.3 Model comparison

Knowing the right model’s structure may make it more interesting to determine its absolute performance. This situation involves model comparison for various defined models (83). Model comparison involves comparing and observing the results of different methods for the same problem. It compares multiple models to prove its performance. Building multiple models for the same problem and comparing their predictions help determine the best model and algorithm for forecasting unnatural outbreaks (13, 25, 34, 42, 46, 47). In general, when assessing model outbreak thresholds, three or more standard deviations from the baseline model can be used. Exponentially weighted moving averages (EWMA) (13) can be used as a method to modify the forecast based on recent errors. Finally, various models are compared to the baseline model to assess its predictive power. Accuracy, recall, F1-score, sensitivity, specificity, ROC, and *p*-value measure model efficacy. When attempting to predict UEs, not all models can be assessed for efficacy using the same set of evaluation indicators. This is where attention must be paid to model comparison for risk assessments of UEs.

## 5 Difference between natural epidemics and UEs in mathematical models and analysis tools

Mathematical models and analysis tools are used for UEs in our review, but they can also be used to determine the occurrence of natural epidemics. The difference exists between natural epidemics and UEs when using mathematical models and analytical tools. The values for natural epidemics and UEs are not the same in the choice of model parameters (60, 62, 63) and warning thresholds (19). However, some studies (13, 20, 44) have also pointed out that some models and tools used for natural epidemics are currently unlikely to provide early detection of UEs. In the current study, data-driven models and mechanistic-based models are less likely to mention the difference between natural epidemics and UEs in terms of model use.

## 6 Mathematical models and analysis tools for risk assessment in various surveillance systems

Several syndrome surveillance systems have been created recently to detect natural epidemics and UEs. Mathematical modeling of surveillance systems has two general directions. One approach simulates various UEs. The second approach is to evaluate warning algorithms for reported event outbreaks using surveillance system data. Currently, the system’s mission is to detect historical outbreaks and determine when the disease will be detected. The systems used in this study are divided into three types: event-based surveillance systems, indicator-based surveillance systems, and hybrid surveillance systems. Supplementary material S7 shows the mathematical models and analysis tools applied in the surveillance systems.

### 6.1 Event-based surveillance systems

Event-based surveillance systems use unstructured data from non-health-sector sources (85). Frequently, data seen daily in society, such as drug sales and absence data, are used as early warning signals (86). It is a common way for such surveillance systems to conduct risk assessment with mathematical models, such as the National Health Service Clinical Assessment System (NHSCAS) and Early Alerting and Reporting (EAR) project. NHSCAS (44, 45) provides early warning of outbreaks caused by UEs or more common infections. The syndrome surveillance system overlays cryptosporidiosis epidemic data onto a statistical model of health hotline (NHS Direct) call data to test it. It calculates the upper limit of prediction for the proportion of diarrhea calls using the confidence interval and control chart methods. A scored risk assessment tool is used in the EAR project (72). It is based on the etiology, health effects, clinical presentation, and epidemiology of the project’s event or outbreak, as well as the sources of information and the potential for intentional release.

### 6.2 Indicator-based surveillance systems

Indicator-based surveillance systems generally analyze data routinely collected from healthcare facilities through institutional disease reporting (87). WSARE algorithm (27, 28) detects outbreak-related anomalies. Date-indexed biomonitoring data (e.g., emergency department data) feeds the algorithm. By comparing date events, statistically significant abnormalities are found and analyzed for early warning. Demographic and hospital monitoring data will alert systems to UEs (19, 20, 29, 30). If a signal exceeds the statistical distortion threshold, an alarm is raised. Commonly used models for this type of system are time series methods (moving average method), CUSUM, and recursive least square adaptive filter. The WHO recommends event-based surveillance to supplement indicator-based surveillance. Indicator-based surveillance may not uncover outbreaks and major public health problems. Additionally, current techniques cannot detect rare but significant outbreaks such as Ebola and avian influenza, as well as novel diseases (86).

### 6.3 Hybrid surveillance systems

Hybrid surveillance systems use both event and indicator variables to construct the model. Mostashari et al. (15) monitor UEs using ambulance dispatch calls. They also employ statistical techniques to measure retrospective data on influenza-like illness (ILI) call types. An alarm was raised when the observed ILI rate exceeded the expected upper confidence limit by 1 day. A system that uses a dengue prediction model using support vector regression (16) relates cases to weather parameters (i.e., temperature, humidity, and rainfall). Even with a small amount of data training, the system can capture dengue trends. The national bioterrorism syndromic surveillance demonstration program (32, 33) combines incidence counts and zip codes to predict the number of episodes of illness using space–time scan statistics. Absenteeism rates (21) are used in system data to track abnormal occurrences. A multi-data surveillance system (18) uses CUSUM and FARR by chief complaints, NHS call types, and the number of records assigned to each syndrome. They detect syndrome numbers rising slowly, acutely, and locally. Time series analysis employed by Early Notification of Community-Based Epidemics (ESSENCE) II (37) for early warning reveals that high-profile events may change detection and alert thresholds.

## 7 Strengths and weaknesses of mathematical models and analysis tools for risk assessment of UEs

### 7.1 Strengths of mathematical models and analysis tools

This study involves two models and a class of tools with established theoretical and computational background. Data-driven models solve biosafety challenges and self-learn for epidemic prediction and monitoring, while mechanistic-based models use extensive knowledge of disease transmission mechanisms to forecast epidemics. This makes disease development more traceable. For decision-makers addressing biosecurity events, the analysis tools in this study are user-friendly and adaptable to multiple diseases. They facilitate quick collaboration among agencies to combat UEs. Machine learning methods are increasingly preferred by researchers in data-driven models. With massive data, machine learning is being applied in UEs. Machine learning enhances tracking and real-time reporting of highly hidden, short-spread, and rapidly spreading unnatural outbreaks.

### 7.2 Weaknesses of mathematical models and analysis tools

Although there are many variations of algorithms in data-driven models in terms of technical means, there are certain requirements for data. The data quality tries to be complete and accurate. If the quality of the data is not satisfactory, it is often necessary to find a way out of the algorithms. Therefore, data-driven models increasingly favor machine learning algorithms. Model parameter fitting makes mechanistic-based model prediction harder. The use of analysis tools, although simple, is generally not superior to the above two types of models in terms of accuracy for event identification.

## 8 Recommendations for the development and applications of mathematical models and analysis tools for risk assessment of UEs

### 8.1 Aspect I: improvements

Mathematical models and analysis tools have advanced, but challenges remain. First, the difficulty of data collection; Complete and accurate data are crucial for data-driven models but limited for UEs. Mechanistic-based models are not influenced by external data, but parameters must be fitted multiple times to improve risk assessment. Second, updating the models is also challenging due to uncertain biological factors. Transmission characteristics of viruses and bacteria need to be accurately identified. Mechanistic-based models struggle with spatial predictions. At the same time, syndromic surveillance solutions typically target one illness. It has a limited ability to distinguish between outbreaks of individual diseases with similar syndromes.

### 8.2 Aspect II: promotional applications

Fully digitized demographic and health data and efficient testing and reporting systems are crucial for effective disease control. Mathematical models and analysis tools play a vital role in controlling epidemics by providing risk assessment. Future applications will focus on improving model parameters to consider social exposure, human activity, economic impact, and environmental impact. Machine learning algorithms will play a significant role in case detection, contact tracking, and intervention responses, narrowing risk assessment to smaller scales and shorter timeframes.

## 9 Conclusion

UEs generate social and work disruptions, mortality, and economic losses due to their covert spread. Current research in this field focuses on analyzing the risk of UEs using symptoms and social data for prediction, early warning, and risk assessment. Mathematical models and analysis tools can be very useful for risk assessment of UEs. Now, using data-driven models combined with non-medical data (such as opinion data) for risk assessment of UEs is indeed becoming more prevalent. Most approaches for differentiating from natural epidemics and UEs globally use analysis tools. However, there are limitations to existing mathematical models and analysis tools, such as limited data availability and the inability to update models in real time. Risk analysis tools rely on data inputs such as data source validity and specified illness parameters of recognized pathogens, which is a limitation. Furthermore, the data used to generate the risk scores was gathered from multiple sources and at various points in time, which may be out of date or contain biases in some circumstances. Data and monitoring errors may also arise during actual events. Most importantly, the included studies, while showing that the models they examined were used for UEs, can also be used to identify the occurrence of natural epidemics. In the current study, mathematical models and analysis tools suggest a distinction between natural epidemics and UEs in the selection of model parameters and warning thresholds.

This review summarizes mathematical models and analysis tools for risk assessment of UEs. If data limitations persist, subsequent model development should focus on model structure or data. For example, the model structure can be improved by replacing the model calculation method to reduce data integrity dependence (e.g., combining a mechanism model with a data-driven approach). In terms of used data, data cleaning can interpolate missing values or consider noisy data. For model factors, the selection will affect the methods of model validation. High-quality surveillance data and indicators are necessary for future surveillance systems. Future model development should consider combining a mechanistic-based model with a data-driven model and learning in the pursuit of time-varying, high-precision risk assessment capabilities.

## Data availability statement

The original contributions presented in the study are included in the article/Supplementary material, further inquiries can be directed to the corresponding authors.

## Author contributions

JL: Conceptualization, Data curation, Formal analysis, Investigation, Writing – original draft, Writing – review & editing. YL: Conceptualization, Data curation, Formal analysis, Investigation, Writing – original draft, Writing – review & editing. ZM: Data curation, Formal analysis, Writing – review & editing. ZL: Data curation, Formal analysis, Writing – review & editing. GZ: Conceptualization, Supervision, Writing – review & editing. CC: Conceptualization, Funding acquisition, Supervision, Writing – review & editing.
