# Supplementary material for: Mathematical models and analysis tools for risk assessment of unnatural epidemics: a scoping review
Source: Front Public Health. 2024 May 2;12:1381328. doi: 10.3389/fpubh.2024.1381328 (PMC11122901; doi:10.3389/fpubh.2024.1381328)

Supplementary Material

**Supplementary Material 1 - Preferred Reporting Items for Systematic reviews and Meta-Analyses extension for Scoping Reviews (PRISMA-ScR) Checklist**

| **SECTION** | **ITEM** | **PRISMA-ScR CHECKLIST ITEM** | **REPORTED ON PAGE #** |
| --- | --- | --- | --- |
| **TITLE** | | | |
| Title | 1 | Identify the report as a scoping review. | 1 |
| **ABSTRACT** | | | |
| Structured summary | 2 | Provide a structured summary that includes (as applicable): background, objectives, eligibility criteria, sources of evidence, charting methods, results, and conclusions that relate to the review questions and objectives. | 1 |
| **INTRODUCTION** | | | |
| Rationale | 3 | Describe the rationale for the review in the context of what is already known. Explain why the review questions/objectives lend themselves to a scoping review approach. | 1-2 |
| Objectives | 4 | Provide an explicit statement of the questions and objectives being addressed with reference to their key elements (e.g., population or participants, concepts, and context) or other relevant key elements used to conceptualize the review questions and/or objectives. | 2 |
| **METHODS** | | | |
| Protocol and registration | 5 | Indicate whether a review protocol exists; state if and where it can be accessed (e.g., a Web address); and if available, provide registration information, including the registration number. | - |
| Eligibility criteria | 6 | Specify characteristics of the sources of evidence used as eligibility criteria (e.g., years considered, language, and publication status), and provide a rationale. | 2-3 |
| Information sources* | 7 | Describe all information sources in the search (e.g., databases with dates of coverage and contact with authors to identify additional sources), as well as the date the most recent search was executed. | 2 |
| Search | 8 | Present the full electronic search strategy for at least 1 database, including any limits used, such that it could be repeated. | 2 |
| Selection of sources of evidence† | 9 | State the process for selecting sources of evidence (i.e., screening and eligibility) included in the scoping review. | 2-3 |
| Data charting process‡ | 10 | Describe the methods of charting data from the included sources of evidence (e.g., calibrated forms or forms that have been tested by the team before their use, and whether data charting was done independently or in duplicate) and any processes for obtaining and confirming data from investigators. | 2-3 |
| Data items | 11 | List and define all variables for which data were sought and any assumptions and simplifications made. | 3 |
| Critical appraisal of individual sources of evidence§ | 12 | If done, provide a rationale for conducting a critical appraisal of included sources of evidence; describe the methods used and how this information was used in any data synthesis (if appropriate). | - |
| Synthesis of results | 13 | Describe the methods of handling and summarizing the data that were charted. | 3 |
| **RESULTS** | | | |
| Selection of sources of evidence | 14 | Give numbers of sources of evidence screened, assessed for eligibility, and included in the review, with reasons for exclusions at each stage, ideally using a flow diagram. | 2-3 |
| Characteristics of sources of evidence | 15 | For each source of evidence, present characteristics for which data were charted and provide the citations. | 3 |
| Critical appraisal within sources of evidence | 16 | If done, present data on critical appraisal of included sources of evidence (see item 12). | - |
| Results of individual sources of evidence | 17 | For each included source of evidence, present the relevant data that were charted that relate to the review questions and objectives. | 3-8 |
| Synthesis of results | 18 | Summarize and/or present the charting results as they relate to the review questions and objectives. | 3-8 |
| **DISCUSSION** | | | |
| Summary of evidence | 19 | Summarize the main results (including an overview of concepts, themes, and types of evidence available), link to the review questions and objectives, and consider the relevance to key groups. | 9-10 |
| Limitations | 20 | Discuss the limitations of the scoping review process. | 10 |
| Conclusions | 21 | Provide a general interpretation of the results with respect to the review questions and objectives, as well as potential implications and/or next steps. | 10 |
| **FUNDING** | | | |
| Funding | 22 | Describe sources of funding for the included sources of evidence, as well as sources of funding for the scoping review. Describe the role of the funders of the scoping review. | 10 |

JBI = Joanna Briggs Institute; PRISMA-ScR = Preferred Reporting Items for Systematic reviews and Meta-Analyses extension for Scoping Reviews.

* Where *sources of evidence* (see second footnote) are compiled from, such as bibliographic databases, social media platforms, and Web sites.

† A more inclusive/heterogeneous term used to account for the different types of evidence or data sources (e.g., quantitative and/or qualitative research, expert opinion, and policy documents) that may be eligible in a scoping review as opposed to only studies. This is not to be confused with *information sources* (see first footnote).

‡ The frameworks by Arksey and O’Malley (6) and Levac and colleagues (7) and the JBI guidance (4, 5) refer to the process of data extraction in a scoping review as data charting*.*

§ The process of systematically examining research evidence to assess its validity, results, and relevance before using it to inform a decision. This term is used for items 12 and 19 instead of "risk of bias" (which is more applicable to systematic reviews of interventions) to include and acknowledge the various sources of evidence that may be used in a scoping review (e.g., quantitative and/or qualitative research, expert opinion, and policy document).

*From:* Tricco AC, Lillie E, Zarin W, O'Brien KK, Colquhoun H, Levac D, et al. PRISMA Extension for Scoping Reviews (PRISMAScR): Checklist and Explanation. Ann Intern Med. 2018;169:467–473. [doi: 10.7326/M18-0850](http://annals.org/aim/fullarticle/2700389/prisma-extension-scoping-reviews-prisma-scr-checklist-explanation).

| **Supplementary Material 2 - Search strategies**  **PubMed:649 results on 31^th^ December 2023** | |
| --- | --- |
| # | **Query** |
| 1 | bioterrorism[MeSH Terms] |
| 2 | biological warfare agents[MeSH Terms] |
| 3 | ((((unnatural epidemic*[Title/Abstract]) OR (unnatural outbreak*[Title/Abstract])) OR (unusual outbreak*[Title/Abstract])) OR (deliberate outbreak*[Title/Abstract])) OR (artificial outbreak*[Title/Abstract]) |
| 4 | ((bioterrorism[Title/Abstract]) OR (biological terrorism[Title/Abstract])) OR (biocrime*[Title/Abstract]) |
| 5 | ((((((((biological warfare agent*[Title/Abstract]) OR (bioterrorism agent*[Title/Abstract])) OR (biological select agent*[Title/Abstract])) OR (biowarfare agent*[Title/Abstract])) OR (biological weapon*[Title/Abstract])) OR (biological warfare*[Title/Abstract])) OR (biological attack*[Title/Abstract])) OR (bio defense*[Title/Abstract])) OR (bio strateg*[Title/Abstract]) |
| 6 subjects | 1 OR 2 OR 3 OR 4 OR 5 |
| 7 | forecasting[MeSH Terms] |
| 8 | risk assessment[MeSH Terms] |
| 9 | ((((forecast*[Title/Abstract]) OR (projection*[Title/Abstract])) OR (predict*[Title/Abstract])) OR (nowcast*[Title/Abstract])) OR (detect*[Title/Abstract]) |
| 10 | ((((risk assessment*[Title/Abstract])) OR (risk analys*[Title/Abstract])) OR (risk identification*[Title/Abstract])) OR (risk evaluation*[Title/Abstract]) |
| 11 | early warning[Title/Abstract] |
| 12 purposes | 7 OR 8 OR 9 OR 10 OR 11 |
| 13 | algorithms[MeSH Terms] |
| 14 | machine learning[MeSH Terms] |
| 15 | (((((model*[Title/Abstract]) OR (simulation*[Title/Abstract])) OR (algorithm*[Title/Abstract])) OR (machine learning[Title/Abstract])) OR (scor*[Title/Abstract]) OR (tool*[Title/Abstract])) |
| 16 methods | 13 OR 14 OR 15 |
| 17 | 6 AND 12 AND 16 (Filters:Humans, English, until 2023/12/31) |

| **Web of Science: 556 results on 31^th^ December 2023** | |
| --- | --- |
| # | **Query** |
| 1 subjects | (((((((((((((((TS=(bioterrorism)) OR TS=(biological warfare agent*)) OR TS=("unnatural epidemic*")) OR TS=("unusual outbreak*")) OR TS=("deliberate outbreak*")) OR TS=("artificial outbreak*")) OR TS=("biological Terrorism")) OR TS=(biocrime*)) OR TS=("bioterrorism agent*")) OR TS=("biological select agent*")) OR TS=("biowarfare agent*")) OR TS=("biological weapon*")) OR TS=("biological warfare*")) OR TS=("biological attack*")) OR TS=("bio defense*")) OR TS=("bio strateg*") |
| 2 purposes | (((((((((TS=(forecast*)) OR TS=(projection*)) OR TS=(predict*)) OR TS=(nowcast*)) OR TS=(detect*)) OR TS=("risk assessment*")) OR TS=("risk analys*")) OR TS=("risk identification*")) OR TS=("risk evaluation*")) OR TS=("early warning") |
| 3 methods | (((((TS=("machine learning")) OR TS=(model*)) OR TS=(simulation*))) OR TS=(algorithm*)) OR TS=(scor*) OR TS=(tool*) |
| 4 | 1 AND 2 AND 3 and Humans(MeSH Headings) and English (Languages) and Article or Review Article (Document Types) |

| **Scopus: 451 results on 31^th^ December 2023** | |
| --- | --- |
| # | **Query** |
| 1 subjects | TITLE-ABS-KEY(bioterrorism OR "biological warfare agent*" OR "unnatural epidemic*" OR "unusual outbreak*"OR "deliberate outbreak*" OR "artificial outbreak*" OR "biological terrorism" OR biocrime* OR "bioterrorism agent*" OR "biological select agent*" OR "biowarfare agent*" OR "biological weapon*" OR "biological warfare*" OR "biological attack*" OR "bio defense*" OR "bio strateg*") |
| 2 purposes | TITLE-ABS-KEY ( forecast* OR projection* OR predict* OR nowcast* OR detect* OR "risk assessment*" OR "risk analys*" OR "risk identification*" OR "risk evaluation*" OR "early warning" ) |
| 3 methods | TITLE-ABS-KEY("machine learning" OR model* OR simulation* OR algorithm* OR scor* OR tool*) |
| 4 | 1 AND 2 AND 3 AND ( LIMIT-TO ( DOCTYPE,"ar" ) OR LIMIT-TO ( DOCTYPE,"re" ) ) AND ( LIMIT-TO ( LANGUAGE,"English" ) ) AND ( LIMIT-TO ( EXACTKEYWORD,"Human" ) OR LIMIT-TO ( EXACTKEYWORD,"Humans" ) ) |

| **Embase: 336 results on 31^th^ December 2023** | |
| --- | --- |
| # | **Query** |
| 1 subjects | 'bioterrorism':ab,kw,ti OR 'biological warfare agent*':ab,kw,ti OR 'unnatural epidemic*':ab,kw,ti OR 'unusual outbreak*':ab,kw,ti OR 'deliberate outbreak*':ab,kw,ti OR 'artificial outbreak*':ab,kw,ti OR 'biological terrorism':ab,kw,ti OR 'biocrime*':ab,kw,ti OR 'bioterrorism agent*':ab,kw,ti OR 'biological select agent*':ab,kw,ti OR 'biowarfare agent*':ab,kw,ti OR 'biological weapon*':ab,kw,ti OR 'biological warfare*':ab,kw,ti OR 'biological attack*':ab,kw,ti OR 'bio defense*':ab,kw,ti OR 'bio strateg*':ab,kw,ti |
| 2 purposes | 'forecast*':ab,kw,ti OR 'projection*':ab,kw,ti OR 'predict*':ab,kw,ti OR 'nowcast*':ab,kw,ti OR 'detect*':ab,kw,ti OR 'risk assessment*':ab,kw,ti OR 'risk analys*':ab,kw,ti OR 'risk identification*':ab,kw,ti OR 'risk evaluation*':ab,kw,ti OR 'early warning':ab,kw,ti |
| 3 methods | 'machine learning':ab,kw,ti OR 'model*':ab,kw,ti OR 'simulation*':ab,kw,ti OR 'algorithm*':ab,kw,ti OR 'scor*':ab,kw,ti OR 'tool*':ab,kw,ti |
| 4 | 1 AND 2 AND 3 AND 'human'/de AND ('article'/it OR 'review'/it) AND [<1966-2023]/py AND [english]/lim |

**Supplementary Material 3 - Study selection form**

**Reviewer,** **date:___________________________________________________________________**

**Number,** **first** **author,**  **year of publication:____________________________________________**

**Review** **summary:**

**Decision** **made** **by** **one** **reviewer** **(after** **fulfilling** **this** **study** **selection** **form)**

Include Ο (all questions below answered “yes”)

Discuss Ο (some question below answered “unknown”)

Exclude Ο (some question below answered “no”)

**Inclusion criteria**

| No | **Criteria** | Yes | No | Unknown |
| --- | --- | --- | --- | --- |
| 1 | Bioterrorism-related terms |  |  |  |
| 2 | Methods applied a model, algorithm, score or simulation |  |  |  |
| 3 | Study objectives included predict, projection, nowcast, detect, forecast, early warning, risk identification, risk evaluation, risk analysis, and risk assessment |  |  |  |
| 4 | Study published in any country |  |  |  |
| 5 | An original article or review |  |  |  |

**Exclusion criteria**

| No. | **Criteria** | Yes | No | Unknown |
| --- | --- | --- | --- | --- |
| 1 | Not humans |  |  |  |
| 2 | Not English language |  |  |  |

**Final** **decision** **made** **by** **the** **reviewers** **(after** **discussion** **with** **other** **reviewer(s))**

Include Ο (all questions below answered “yes”)

Exclude Ο (some question below answered “no”), reason number:__________________________

**Supplementary Material 4 - Data extraction form**

**Reviewer (name,date):________________________________________________________**

**Study number, from list:______________________________________________________**

| **Basic information of the article** | |
| --- | --- |
| No. | Indicators |
| 1 | Year of publication |
| 2 | Country |
| 3 | First author |
| 4 | Journal |
| **Data extraction** | |
| No. | Indicators |
| 1 | Name of model |
| 2 | Data type |
| 3 | Data (time or events) |
| 4 | Functions |
| 5 | Factors |
| 6 | Outcomes |
| 7 | Name of system |
| 8 | Model in system |
| 9 | Model function (alarm threshold) |

**Final** **decision** **made** **by** **the** **reviewers** **(after** **discussion** **with** **other** **reviewer(s))**

Include Ο (all questions below answered “Indicators can be extracted”)

Exclude Ο (some question below answered “Indicators can not be extracted”), reason number:__________________________

**Supplementary Material 5 - General characteristics of the published research papers for risk assessment of unnatural epidemics (N=66)**

| Characteristics | Studies, n (%) |
| --- | --- |
| **Year of publication** | |
| 2001 | 1 (1.5) |
| 2002 | 4 (6.1) |
| 2003 | 11 (16.7) |
| 2004 | 8 (12.1) |
| 2005 | 6 (9.1) |
| 2006 | 3 (4.5) |
| 2007 | 3 (4.5) |
| 2008 | 3 (4.5) |
| 2009 | 5 (7.6) |
| 2010 | 4 (6.1) |
| 2012 | 1 (1.5) |
| 2013 | 3 (4.5) |
| 2014 | 1 (1.5) |
| 2015 | 1 (1.5) |
| 2016 | 3 (4.5) |
| 2017 | 1 (1.5) |
| 2018 | 2 (3.0) |
| 2019 | 1 (1.5) |
| 2020 | 1 (1.5) |
| 2021 | 2 (3.0) |
| 2022 | 1 (1.5) |
| 2023 | 1 (1.5) |
| **Country of first author** | |
| United States | 41 (62.1) |
| Serbia | 4 (6.1) |
| United Kingdom | 3 (4.5) |
| Japan | 3 (4.5) |
| Germany | 2 (3.0) |
| Australia | 2 (3.0) |
| Belarus | 1 (1.5) |
| Canada | 1 (1.5) |
| China | 1 (1.5) |
| France | 1 (1.5) |
| Greece | 1 (1.5) |
| Hungary | 1 (1.5) |
| Israel | 1 (1.5) |
| Italy | 1 (1.5) |
| Korea | 1 (1.5) |
| Pakistan | 1 (1.5) |
| Sweden | 1 (1.5) |
| **Journal** | |
| Emerging Infectious Diseases | 6 (9.1) |
| MMWR Supplements | 6 (9.1) |
| Risk Analysis | 6 (9.1) |
| Epidemiology and Infection | 4 (6.1) |
| Journal of the American Medical Informatics Association | 4 (6.1) |
| Biosecurity and Bioterrorism: Biodefense Strategy, Practice, and Science | 3 (4.5) |
| BMC Medical Informatics and Decision Making | 3 (4.5) |
| Journal of Urban Health: Bulletin of the New York Academy of Medicine | 3 (4.5) |
| Statistics in Medicine | 3 (4.5) |
| Military Medicine | 2 (3.0) |
| Public Health | 2 (3.0) |
| AIHA Journal | 1 (1.5) |
| American Journal of epidemiology | 1 (1.5) |
| AMIA 2002 Annual Symposium Proceedings | 1 (1.5) |
| AMIA 2003 Symposium Proceedings | 1 (1.5) |
| AMIA 2009 Symposium Proceedings | 1 (1.5) |
| Analytical Sciences | 1 (1.5) |
| Central European Journal of Public Health | 1 (1.5) |
| Clinical Microbiology and Infection | 1 (1.5) |
| Environmental Health and Preventive Medicine | 1 (1.5) |
| Environmental Science and Technology | 1 (1.5) |
| Epidemics | 1 (1.5) |
| Eurosurveillance | 1 (1.5) |
| Health Security | 1 (1.5) |
| Israel Medical Association Journal | 1 (1.5) |
| Japanese Society of Chemotherapy and The Japanese Association for Infectious Diseases | 1 (1.5) |
| JMIR public health and suirveillance | 1 (1.5) |
| Journal of Biomedical Informatics | 1 (1.5) |
| Journal of Infection and Public Health | 1 (1.5) |
| Journal of public health management and practice | 1 (1.5) |
| Mathematical Biosciences and Engineering | 1 (1.5) |
| Open Medicine | 1 (1.5) |
| Plos Computational Biology | 1 (1.5) |
| Proceedings of the National Academy of Sciences of the United States of America | 1 (1.5) |
| Public Health Reports | 1 (1.5) |

**Supplementary Material 6 - Articles published in risk assessment research based on mathematical models and analysis tools**

| **No.** | **Year of publication** | **Country of first author** | **First author** | **Journal** |
| --- | --- | --- | --- | --- |
| 1 | 2001 | United States | Meltzer MI | Emerging Infectious Diseases |
| 2 | 2002 | United States | Nicas M | Risk analysis |
| 3 | 2002 | United States | Tsui FC | AMIA 2002 Annual Symposium Proceedings |
| 4 | 2002 | United States | Rotz LD | Emerging Infectious Diseases |
| 5 | 2002 | Germany | Grunow R | Clinical Microbiology and Infection |
| 6 | 2003 | Belarus | Reshetin VP | Risk analysis |
| 7 | 2003 | United States | Mostashari F | Journal of Urban Health: Bulletin of the New York Academy of Medicine |
| 8 | 2003 | United States | Nicas M | AIHA Journal |
| 9 | 2003 | United States | Treadwell TA | [Public Health Reports](https://journals.sagepub.com/home/PHR) |
| 10 | 2003 | United States | Lombardo J | Journal of Urban Health: Bulletin of the New York Academy of Medicine |
| 11 | 2003 | United States | Gesteland PH | Journal of the American Medical Informatics Association |
| 12 | 2003 | United States | Reis BY | AMIA 2003 Symposium Proceedings |
| 13 | 2003 | United States | Tsui FC | Journal of the American Medical Informatics Association |
| 14 | 2003 | United States | Wong WK | Journal of Urban Health: Bulletin of the New York Academy of Medicine |
| 15 | 2003 | United States | Reis BY | BMC Medical Informatics and Decision Making |
| 16 | 2003 | United States | Reis BY | Proceedings of the National Academy of Sciences of the United States of America |
| 17 | 2004 | United States | Fennelly KP | Emerging Infectious Diseases |
| 18 | 2004 | United States | Walden J | Emerging Infectious Diseases |
| 19 | 2004 | United States | Wallenstein S | MMWR supplements |
| 20 | 2004 | United States | Kleinman K | American Journal of epidemiology |
| 21 | 2004 | United States | Dembek ZF | MMWR supplements |
| 22 | 2004 | United States | Yih WK | MMWR supplements |
| 23 | 2004 | United States | Yuan CM | MMWR supplements |
| 24 | 2004 | France | Legrand J | Epidemiology and Infection |
| 25 | 2005 | United States | Brillman JC | BMC Medical Informatics and Decision Making |
| 26 | 2005 | United States | Buckeridge DL | MMWR supplements |
| 27 | 2005 | United States | Lawson BM | Journal of public health management and practice |
| 28 | 2005 | United States | Nordin JD | Emerging Infectious Diseases |
| 29 | 2005 | United States | Kleinman KP | MMWR supplements |
| 30 | 2005 | Japan | Ohkusa Y | Japanese Society of Chemotherapy and The Japanese Association for Infectious Diseases |
| 31 | 2006 | United Kingdom | Cooper DL | Epidemiology and Infection |
| 32 | 2006 | United Kingdom | Smith GE | Eurosurveillance |
| 33 | 2006 | United States | Dembek ZF | Epidemiology and Infection |
| 34 | 2007 | Israel | Kaufman Z | Israel Medical Association Journal |
| 35 | 2007 | United States | Kulldorff M | Statistics in Medicine |
| 36 | 2007 | United States | Hogan WR | Statistics in Medicine |
| 37 | 2008 | United States | Murphy SP | Journal of the American Medical Informatics Association |
| 38 | 2008 | United States | Shen Y | Journal of Biomedical Informatics |
| 39 | 2008 | Sweden | Meyer N | Epidemiology and Infection |
| 40 | 2009 | United States | Legrand J | Plos Computational Biology |
| 41 | 2009 | Japan | Kobari T | Analytical Sciences |
| 42 | 2009 | Serbia | Radosavljevic V | [Biosecurity and Bioterrorism: Biodefense Strategy, Practice, and Science](https://www.liebertpub.com/journal/bsp) |
| 43 | 2009 | Canada | Izadi M | AMIA 2009 Symposium Proceedings |
| 44 | 2009 | Greece | Pappas G | Journal of Infection and Public Health |
| 45 | 2010 | United Kingdom | Egan JR | Epidemics |
| 46 | 2010 | United States | Ray J | Statistics in Medicine |
| 47 | 2010 | United States | McBrien KA | BMC Medical Informatics and Decision Making |
| 48 | 2010 | Japan | Zenihana T | Environmental Health and Preventive Medicine |
| 49 | 2012 | Serbia | Radosavljevic V | Public Health |
| 50 | 2013 | United States | Del Valle SY | Mathematical Biosciences and Engineering |
| 51 | 2013 | United States | Cheng KE | Journal of the American Medical Informatics Association |
| 52 | 2013 | Germany | Tomuzia K | Biosecurity and Bioterrorism: Biodefense Strategy, Practice, and Science |
| 53 | 2014 | Italy | Riccardo F | Biosecurity and Bioterrorism: Biodefense Strategy, Practice, and Science |
| 54 | 2015 | United States | Hong T | Environmental Science and Technology |
| 55 | 2016 | Pakistan | Ali MA | Public Health |
| 56 | 2016 | Serbia | Radosavljevic V | Central European Journal of Public Health |
| 57 | 2016 | Korea | Rhee C | Health Security |
| 58 | 2017 | United States | Rainisch G | Emerging Infectious Diseases |
| 59 | 2018 | United States | McClellan G | Risk analysis |
| 60 | 2018 | United States | Cieslak TJ | Military Medicine |
| 61 | 2019 | Australia | Chen X | Risk analysis |
| 62 | 2020 | Australia | Chen X | Risk analysis |
| 63 | 2021 | Serbia | Radosavljevic V | [Open Medicine](https://letpub.com.cn/index.php?page=journalapp&view=detail&journalid=10184) |
| 64 | 2021 | United States | Miller M | JMIR public health and suirveillance |
| 65 | 2022 | China | Lin M | Risk analysis |
| 66 | 2023 | Hungary | [Farkas](https://pubmed.ncbi.nlm.nih.gov/?term=Farkas+CB&cauthor_id=35569934) CB | Military Medicine |

**Supplementary Material 7 - Mathematical models and analysis tools for risk assessment in various surveillance system**

| **Type of system** | **Name of system** | **Model** | **Alarm threshold** |
| --- | --- | --- | --- |
| Event-based | NHS* Clinical Assessment System (45,86) | Control chart method and Confidence interval method | The proportion of diarrhoea calls exceeded the upper 99.5% control chart limit or upper 99.5% confidence interval. |
|  | Early Alerting and Reporting project (71) | Risk assessment matrix | Items scored over 14, the issue of an alert was suggested. |
| Indicator  -based | Military Active Real-time Syndromic Surveillance system (19) | CUSUM* | If a signal exceeds the statistical distortion threshold, an alarm is raised. |
|  | Hospital Admissions Syndromic Surveillance (20) | Moving average method | Identify differences of >3 standard deviations for each syndrome from a 6-month moving average. |
|  | Israel Center for Disease  Control Surveillance System (28,29) | WSARE* algorithm | Detect anomalous pattern. |
|  | Real-time Outbreak and Disease Surveillance System (30,31) | Recursive least square adaptive filter | Trigger a signal alarm when the current actual count exceeded the 95% confidence interval of its predicted count. |
| Hybrid | Computerized Ambulance Dispatch System (15) | Modified cyclical regression model | A day when the observed ILI* rate exceeded the expected upper confidence limit. |
|  | ID-Viewer (16) | Support vector regression | NA* |
|  | Multi-data Surveillance System (18) | CUSUM* and FARR* | A 'signal' was triggered in the system when the cumulated differences or significant increase in observed and expected counts exceeded a predetermined threshold. |
|  | Early Aberration Reporting System (21) | Moving average method | Identify differences of >3 standard deviations for each syndrome from a 6-month moving average. |
|  | National Bioterrorism Syndromic Surveillance Demonstration Program (33,34) | Space-time scan statistic | If a signal exceeds the statistical distortion threshold, an alarm is raised. |
|  | ESSENCE* II (38) | Modified exponentially weighted moving average technique | The occurrence of high-profile events may change detection and alerting thresholds. |

*The following abbreviations are used. NHS: National health service; WSARE: What’s strange about recent events; CUSUM: Cumulative sum; ILI: Influenzalike illness; ESSENCE II: Early notification of community-based epidemics II; FARR: Exceedance method by Farrington; ID: Infectious disease; NA: No answer, used on forms.

**Supplementary Material 8 - Graphical abstract**


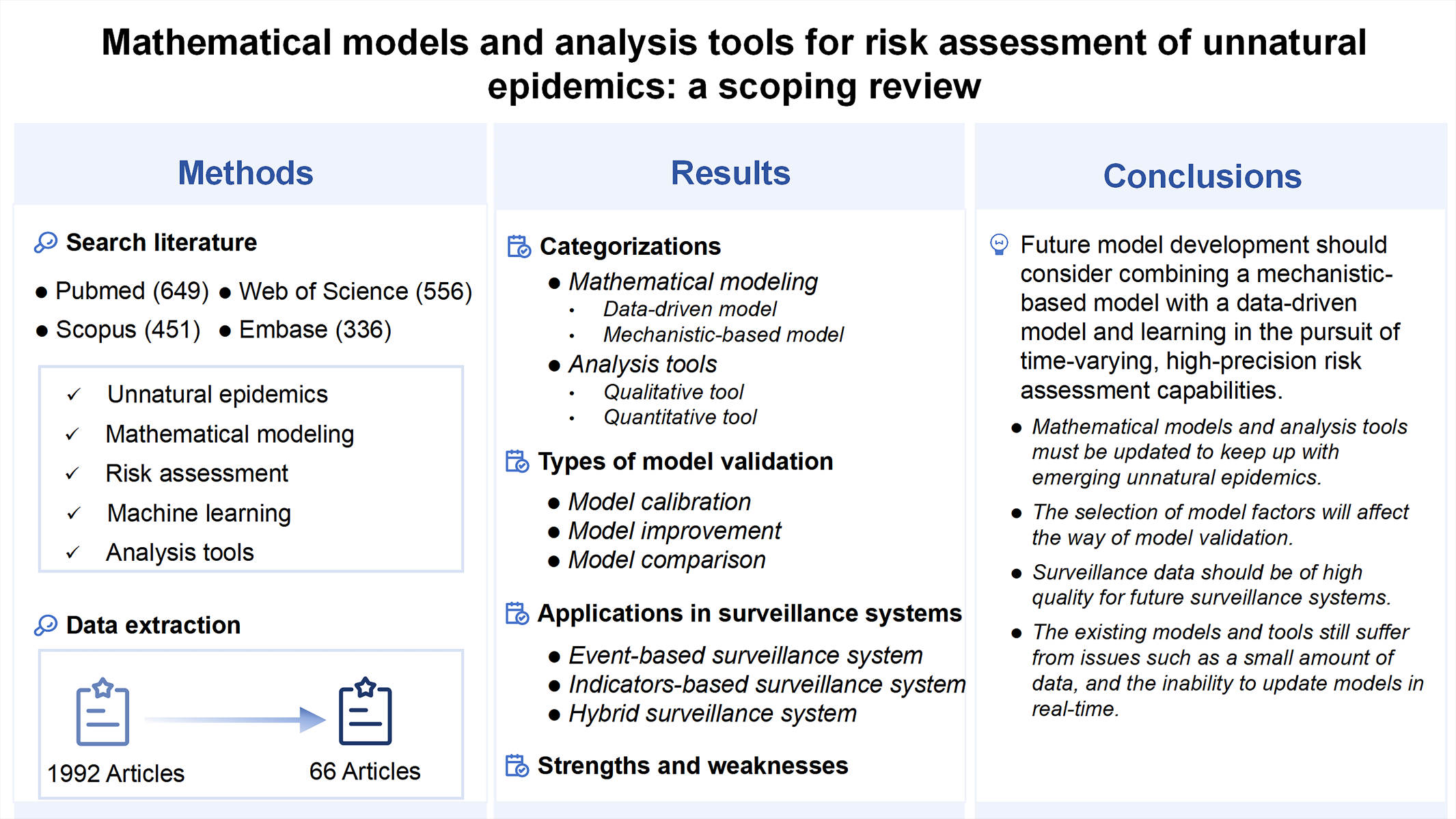

Supplement: Supplementary file 1 [file Table_1.DOCX]
